# Beneficial Effect of Low-Fat Elemental Diet Therapy on Pain in Chronic Pancreatitis

**DOI:** 10.1155/2014/862091

**Published:** 2014-04-14

**Authors:** Tsukasa Ikeura, Makoto Takaoka, Kazushige Uchida, Hideaki Miyoshi, Kazuichi Okazaki

**Affiliations:** The Third Department of Internal Medicine, Kansai Medical University, 2-3-1 Shinmachi, Hirakata, Osaka 573-1191, Japan

## Abstract

*Background & Aims*. Chronic pancreatitis (CP) is often associated with abdominal pain, which impairs quality of life. The aim of this prospective study was to clarify whether the use of a low-fat elemental diet (ED) is beneficial for managing pain in patients with CP. *Methods*. Seventeen CP patients with pain despite fat-restricted dietary and conventional medical treatments were enrolled in this prospective study. These patients received low-fat ED therapy in addition to restricting fat intake for 8 weeks. The change of pain severity was examined by interviewing the patient and status of analgesic use. *Results*. Mean serum levels of amylase and lipase at 8 weeks after the beginning of low-fat ED therapy decreased compared to those before the therapy. At 8 weeks, pain alleviation after low-fat ED therapy was reported in 15 out of 17 patients (88%). Of these 15 patients, 10 patients reported complete pain disappearance. One of 3 patients with severe or moderate pain requiring analgesic was relieved of analgesic use after low-fat ED therapy. *Conclusion*. Low-fat ED therapy is useful as means of pain control in CP. The therapy is recommended in outpatients with CP who present with pain despite conventional dietary and medical treatments.

## 1. Introduction

Chronic pancreatitis (CP) is characterized histologically by the destruction of acinar cells and presence of irregular fibrosis within the pancreas. Most patients with CP have abdominal pain, maldigestion, and diabetes mellitus [1, 2]. The most frequent and serious clinical symptom is abdominal pain, which occurs in at least 75% of patients suffering from CP [3]. The recurrent or protracted abdominal pain in CP is often difficult to manage and decreases patients' quality of life. Pain management in CP includes dietary treatment (e.g., fat restriction and cessation of alcohol and tobacco use), medical treatment (e.g., analgesics, high-dose pancreatic enzymes, and acid suppression agents), endoscopic treatment (e.g., sphincterotomy, lithotripsy, and pancreatic duct stenting), and surgery. American Gastroenterological Association guidelines recommend a low-fat diet as the initial management for the pain in CP [4].

It is well known that an elemental diet (ED) has a protective effect in patients suffering from Crohn's disease [5]. ED has the following features: (1) the digestion of protein by pancreatic enzymes is not required because nitrogen is derived from amino acids alone; (2) ED contains minimal fat. A recent paper by Ito et al. showed 2 patients in whom the oral administration of a low-fat ED (Elental; Ajinomoto Pharmaceutical Ltd., Tokyo, Japan) was useful for alleviating pain in CP [6]. We conjecture that this beneficial effect results from “pancreas rest” due to the extremely small amount of fat in the diet.

To the best of our knowledge, no additional survey has confirmed the utility of low-fat ED therapy in CP patients with pain. The aim of this prospective study was to clarify whether the use of a low-fat ED is beneficial for managing pain in patients with CP.

## 2. Materials and Methods

### 2.1. Patients

Patients with CP who presented with pain despite fat-restricted dietary and conventional medical treatments were enrolled in this prospective study between 2009 and 2012. Patients who had undergone past endoscopic and surgical interventions were excluded from this study. All patients underwent routine laboratory tests and imaging studies including ultrasonography, computed tomography, and magnetic resonance cholangiopancreatography. In some patients, additional examinations such as pancreatic exocrine function test (the* N*-benzoyl-L-tyrosyl-p-aminobenzoic acid test) [7], endoscopic ultrasonography, and endoscopic retrograde cholangiopancreatography were performed. Using the findings of these examinations, the diagnosis of CP (definitive, probable, early, and possible) was established according to the clinical diagnostic criteria proposed by the Japan Pancreas Society [8].

### 2.2. Study Protocol

After obtaining informed consent, their clinical data (sex, age, etiology of CP, smoking habit, and diabetes status) were recorded. Information about concurrent medications such as analgesics, pancreatic enzymes, acid suppression agents, and protease inhibitors was also collected.

Low-fat ED therapy was performed on an outpatient basis using Elental, which is commercially available in Japan. This powdered formula contains 79.3% dextrin, 17.6% amino acids, 0.6% soybean oil, 2.0% minerals, and 0.5% vitamins, by weight (Table 1) [9, 10]. The patients were instructed to take at least 1 packet (80 g) of Elental per day in addition to restricting their fat intake. A packet of Elental was dissolved in 300 mL of lukewarm water for an eventual concentration of 1 kcal/mL.

Pain severity was assessed at 0 weeks (at the beginning of the low-fat ED therapy) and at 4 and 8 weeks (after the initiation of therapy) by performing patient interviews and assessing the status of analgesic use. Pain degree was graded according to 3 levels as follows: grade 2, severe or moderate pain requiring analgesics; grade 1, mild pain not requiring analgesics; and grade 0, no pain. Moreover, we determined the serum levels of amylase, lipase, total protein, and albumin and evaluated the adverse events at 0, 4, and 8 weeks.

### 2.3. Definitions

CP was classified into 2 distinct categories according to etiology: alcoholic and nonalcoholic. Alcoholic CP was diagnosed in patients with a daily intake of pure alcohol >80 g for at least 5 years. Nonalcoholic CP included idiopathic CP and CP induced by other etiological factors, including neoplasm and congenital anomaly [7]. The diagnosis of diabetes was defined as a fasting glucose level >127 mg/dL (7 mmol/L) or a glucose level >201 mg/dL (11 mmol/L) 2 hours after a glucose load [11].

### 2.4. Statistical Analysis

Differences among groups were analyzed using Student's *t*-test for quantitative variables. A *P* value <0.05 was considered statistically significant.

## 3. Results

Nineteen patients with CP were prospectively registered in this study. However, 2 patients were excluded from the study: 1 patient with diabetes showed aggravated glycemic control at 4 weeks after the initiation of ED therapy, and another patient was lost to follow-up. Accordingly, a total of 17 patients (4 males and 13 females; mean age, 49.8 ± 11.7 years) were studied (Table 2). Alcoholic and nonalcoholic CP were observed in 4 patients (24%) and 13 patients (76%), respectively. The etiologies of nonalcoholic CP were idiopathic in 11 patients (65%), pancreas divisum in 1 patient (6%), and intraductal papillary mucinous neoplasm in 1 patient (6%). Two patients (12%) were diagnosed as definitive CP and 15 patients (88%) as probable CP according to the Japanese diagnostic criteria for CP. Prior to the initiation of low-fat ED therapy, 15 patients (88%) were treated with other medical therapies for pain, including acid suppression agents in 13 patients (76%), pancreatic enzymes in 10 patients (59%), protease inhibitor, that is, camostat mesilate, in 9 patients (53%), and analgesics in 3 patients (18%). Diabetes was observed in 1 patient (6%), who was treated with glimepiride. One packet (80 g) of Elental per day was taken by 10 patients (59%), 2 packets (160 g) by 3 patients (18%), and 3 packets (240 g) by 4 patients (23%).

**Figure 1:**
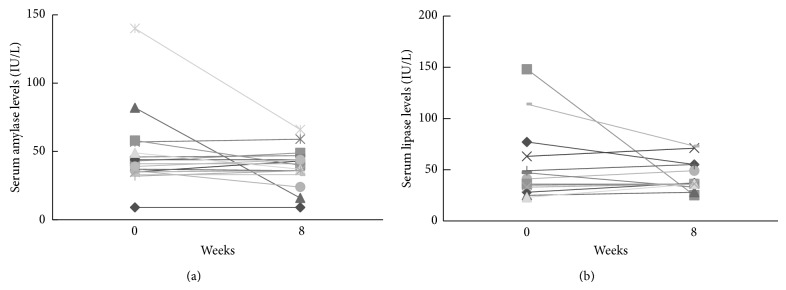
Serum levels of amylase and lipase at 0 and 8 weeks after low-fat ED therapy. (a) Amylase. (b) Lipase.

Mean serum levels of amylase and lipase at 8 weeks after low-fat ED therapy decreased compared to those at 0 weeks (amylase, 48 ± 27.2 IU/L at 0 weeks versus 38.8 ± 13.5 IU/L at 8 weeks; lipase, 55.3 ± 36 IU/L at 0 weeks versus 43.8 ± 14.9 IU/L at 8 weeks), but the differences were not significant (Figure 1).

**Figure 2:**
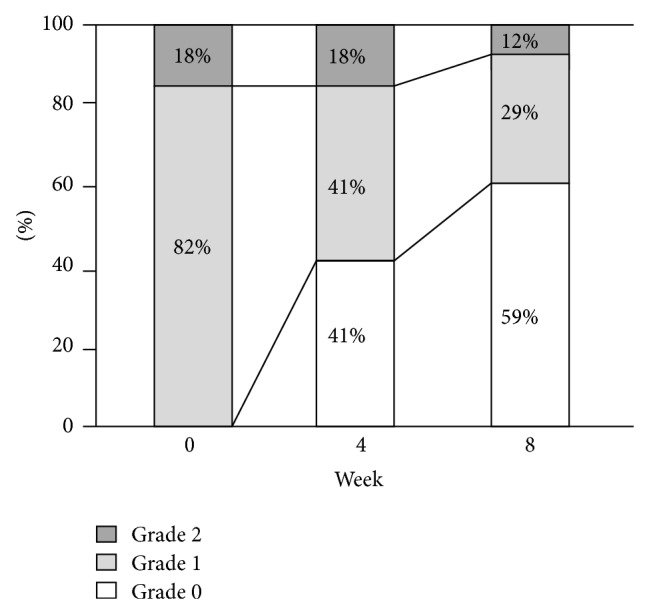
Evaluation of pain using pain grades at 0, 4, and 8 weeks.

At 8 weeks, pain alleviation after the low-fat ED therapy was reported in 15 of 17 patients (88%). Of these 15 patients, 10 patients reported complete pain disappearance. One of 2 patients whose pain did not improve had calcified CP accompanied with pancreatic atrophy indicative of the so-called late-stage CP. No patient experienced pain exacerbation after the low-fat ED therapy. The evaluation of pain using pain grades at 0, 4, and 8 weeks is shown in Figure 2. At the beginning of the low-fat ED therapy, pain severity was classified as grade 2 in 3 patients (18%) and grade 1 in 14 patients (82%). At 8 weeks, the number of patients with grade 2 and grade 1 decreased to 2 patients (12%) and 5 patients (29%), respectively.

Patients' mean serum levels of total protein and albumin at 8 weeks did not differ significantly from those at 0 weeks (total protein, 73.5 ± 5.5 g/L at 0 weeks versus 73.4 ± 5.1 g/L at 8 weeks; albumin, 46.3 ± 3.0 g/L at 0 weeks versus 44.4 ± 3.1 g/L at 8 weeks).

## 4. Discussion

CP is often associated with recurrent or protracted pain that impairs quality of life [1–3]. The pathogenesis of pain generation in CP remains incompletely understood despite considerable research [12]. The most potential source of pain in CP is increased pressure within the pancreatic ducts and tissues resulting from the hyperstimulation of exocrine pancreatic secretion, pancreatic stones, or strictures of the pancreatic duct [13, 14]. Fat intake induces the release of cholecystokinin (CCK) in the duodenum, which stimulates secretions from the pancreatic exocrine gland [15]. Therefore, it is theoretically reasonable to restrict oral fat intake to prevent painful relapses. Indeed, a low-fat enteral supplement has been shown to minimally increase plasma CCK levels, leading to reduced postprandial pain in CP [16].

ED is a digested nutritional supplement that consists of amino acids, dextrin or glucose, and minimal fat. A few reports have shown that ED was useful in the management of pancreatitis since it is less simulating to the pancreas [5, 17, 18]. In particular, the Elental preparation used in the present study contains an extremely small amount of fat (0.17 g/100 kcal). Pain reduction was seen in 88% of CP patients on low-fat ED therapy in addition to dietary fat restriction. Moreover, 1 of 3 patients with severe or moderate pain requiring an analgesic was relieved of the analgesic use after low-fat ED therapy. Although the precise mechanism by which low-fat ED therapy exerts the therapeutic efficacy for pain in CP is unknown, it is postulated that this therapy may enhance the efficacy of restricting fat intake in meals and reduce pain by decreasing the release of CCK.

Enteral nutrition therapy using ED was reported to have supportive effects in patients with Crohn's disease, including the remission of active disease, maintaining the remission of quiescent disease, and preventing postoperative recurrence [5, 19]. These supportive effects are thought to be associated with improved bacterial flora, low antigenicity, and improved nutritional status [20, 21]. Meanwhile, some reports have suggested that amino acids such as glutamine, glycine, and histamine have an anti-inflammatory effect on animal experimental and human chronic inflammatory disease [22–26]. Therefore, the abundant amino acids within ED might directly alleviate pancreatic inflammation and lead to pain relief. This hypothesis has yet to be verified.

Nutrition support is considered important in the management of CP because patients suffering from CP sometimes experience maldigestion due to pancreatic exocrine insufficiency resulting from ongoing pancreatic destruction [1]. Another advantage of ED is that the formula consists mainly of amino acids and dextrin, which do not require pancreatic enzymes for digestion and absorption. Therefore, ED is expected to offer optimal nutrition support even in patients with CP who have severe exocrine insufficiency. In this study, the use of ED had no influence on nutritional parameters such as serum levels of total protein and albumin. The reason for this is that most of the patients included in this study had good nutrition because of pancreatic exocrine sufficiency or the administration of pancreatic enzymes.

Ito et al. reported 2 cases of calcified CP with repeated pain episodes that could be alleviated using low-fat ED therapy [6]. In this study, only 12% of the patients had calcified CP, which was diagnosed as definite CP according to Japanese diagnostic criteria. Pancreatic pain tends to be seen more frequently in the early stage rather than in the late stage of CP [27]. Since the pain observed in patients with late-stage CP is frequently caused by pancreatic stones, endoscopic and surgical intervention is often required. The results of this study indicate that low-fat ED therapy is more effective for patients with early-stage CP than for patients with late-stage CP.

To date, several attempts have been made to evaluate the degree of pain in CP using different pain scales and protocols (e.g., a visual analogue scale) [28, 29]. However, it is difficult to accurately evaluate pain intensity since the individual perception of pain varies among patients. A standard means for evaluating the utility of pain treatment is still lacking [28]. In this study, in addition to interviewing each patient, the pain degree was classified into 3 grades based on the presence or absence of analgesics use to make it easier to evaluate the degree of pain.

Low-fat ED therapy is safe and simple. However, a patient suffering from diabetes had to abandon ED therapy at 4 weeks due to aggravated glycemic control. Particular attention is needed when administering ED therapy to patients with CP and diabetes.

In summary, low-fat ED therapy might be useful for pain management of outpatients with CP when patients experience acute and recurring pain attacks. The results of this study need to be confirmed by a randomized study comparing pain control responses between patients with CP who are or are not treated with low-fat ED therapy.

## Figures and Tables

**Table 1:** Ingredients of Elental (total 100 g).

Amino acids	17.6 g
L-Isoleucine	803 mg
L-Leucine	1124 mg
L-Lysine-HCl	1110 mg
L-Methionine	810 mg
L-Phenylalanine	1089 mg
L-Threonine	654 mg
L-Tryptophan	189 mg
L-Valine	876 mg
L-Histidine-HCl-H_2_O	626 mg
L-Arginine-HCl	1406 mg
L-Alanine	1124 mg
Mg-K-L-Aspartate	1295 mg
Na-L-Aspartate-H_2_O	1084 mg
L-Glutamine	2415 mg
Glycine	631 mg
L-Proline	788 mg
L-Serine	1449 mg
L-Tyrosine	138 mg
Carbohydrate (dextrin)	79.3 g
Lipid (soybean oil)	0.6 g
Vitamin A	810 IU
Vitamin D	64 IU
Vitamin B1	0.24 mg
Vitamin B2	0.25 mg
Vitamin B6	0.33 mg
Niacin	2.78 mg
Pantothenic acid	1.38 mg
Folic acid	55 *μ*g
Vitamin B12	0.88 *μ*g
Vitamin C	9.75 mg
Vitamin K	11.3 *μ*g
Vitamin E	4.13 IU
Biotin	48.8 *μ*g
Choline	10.7 mg
Na	325 mg
K	272 mg
Cl	646 mg
Mg	50 mg
Ca	197 mg
P	152 mg
Fe	2.25 mg
I	19 *μ*g
Mn	375 *μ*g
Cu	250 *μ*g
Zn	2.25 mg

**Table 2:** Characteristics of analyzed patients.

	All patients (*n* = 17)
Gender	
Male	4 (24%)
Female	13 (76%)
Age (mean ± SD)	49.8 ± 11.7
Smokers	3 (18%)
Etiology	
Nonalcoholic	13 (76%)
Alcoholic CP	4 (24%)
Diagnosis by Japanese diagnostic criteria	
Definitive CP	2 (12%)
Possible CP	15 (88%)
Medication	
Acid suppression agents	13 (76%)
Pancreatic enzymes	10 (59%)
Protease inhibitors	9 (53%)
Analgesics	3 (18%)
Diabetes	1 (6%)
Dosage amount of Elental per day	
80 g	10 (59%)
160 g	3 (18%)
240 g	4 (23%)
